# A Pillar-Free Diffusion Device for Studying Chemotaxis on Supported Lipid Bilayers

**DOI:** 10.3390/mi12101254

**Published:** 2021-10-16

**Authors:** Jia Hao, Winfield Zhao, Jeong Min Oh, Keyue Shen

**Affiliations:** 1 Department of Biomedical Engineering, University of Southern California, Los Angeles, CA 90089, USA; jiahao@usc.edu (J.H.); wtzhao@usc.edu (W.Z.); ohjeongm@usc.edu (J.M.O.); 2 Norris Comprehensive Cancer Center, University of Southern California, Los Angeles, CA 90033, USA; 3 USC Stem Cell, University of Southern California, Los Angeles, CA 90033, USA

**Keywords:** chemotaxis, micro-milling, microdevice, supported lipid bilayer, membrane-bound interactions, ICAM-1, CXCL12

## Abstract

Chemotactic cell migration plays a crucial role in physiological and pathophysiological processes. In tissues, cells can migrate not only through extracellular matrix (ECM), but also along stromal cell surfaces via membrane-bound receptor–ligand interactions to fulfill critical functions. However, there remains a lack of models recapitulating chemotactic migration mediated through membrane-bound interactions. Here, using micro-milling, we engineered a multichannel diffusion device that incorporates a chemoattractant gradient and a supported lipid bilayer (SLB) tethered with membrane-bound factors that mimics stromal cell membranes. The chemoattractant channels are separated by hydrogel barriers from SLB in the cell loading channel, which enable precise control of timing and profile of the chemokine gradients applied on cells interacting with SLB. The hydrogel barriers are formed in pillar-free channels through a liquid pinning process, which eliminates complex cleanroom-based fabrications and distortion of chemoattractant gradient by pillars in typical microfluidic hydrogel barrier designs. As a proof-of-concept, we formed an SLB tethered with ICAM-1, and demonstrated its lateral mobility and different migratory behavior of Jurkat T cells on it from those on immobilized ICAM-1, under a gradient of chemokine CXCL12. Our platform can thus be widely used to investigate membrane-bound chemotaxis such as in cancer, immune, and stem cells.

## 1. Introduction

Cell migration plays a crucial role in physiological and pathophysiological processes, such as tissue regeneration [1,2], immunosurveillance [3,4], and cancer metastasis [5,6,7]. During cell migration, cells typically respond to a gradient of chemoattractants, and migrate through the extracellular matrix (ECM). Notably, cells can also migrate along the surfaces of surrounding stromal cells in tissue microenvironments through membrane-bound receptor–ligand interactions. For instance, homing and engraftment of hematopoietic stem cells (HSCs) involves complex interactions of HSCs with vascular endothelial cells, macrophages, and mesenchymal stromal cells through a range of cell–cell adhesion molecules on both stem and stromal cells [8] and chemokines [9]. During immunosurveillance, circulating T cells exit and return to the blood circulation through T cell interactions with vascular/lymphatic endothelial cells [10]. They also migrate over the surface of antigen presenting cells and other somatic cells in search for pathogenic antigens [11]. In cancer metastasis, cancer cells often lodge in the microvasculature in distant organs and transmigrate through the endothelium via direct cell–cell adhesion to form micrometastases [12,13]. It is thus important to understand chemotaxis in the context of membrane-bound interactions.

However, there remains a lack of models recapitulating chemotactic migration in such context. The existing in vitro platforms designed for chemotactic studies, including Boyden chamber and microfluidic assays, have largely been designed for cell migration on ECM [14,15]. Some studies investigating the role of membrane-bound factors, such as ICAM-1, have also been modeled as immobilized factors on solid substrates [16,17], which lacks the unique properties associated with their membrane-bound natures such as lateral mobility and molecular orientation [18]. Recently, researchers have developed cellular cultures in microfluidic channels to directly evaluate cell migration through stromal cells (often endothelial cells) [19,20,21,22]. However, such studies often lack a clearly defined chemoattractant and/or involve multiple membrane-bound interactions that are difficult to delineate in the coculture systems.

Supported lipid bilayers (SLBs) tethered with biomolecules have been widely adopted as a model of cell membranes, with extraordinary success in studying immune cell activation and stem cell niche interactions [23,24,25]. However, cell migration in response to a chemoattractant gradient on SLBs tethered with membrane-bound factors, to the best of our knowledge, has not been reported. There remains a lack of models that incorporate both chemoattractant gradient and SLBs in the same system, partly due to the delicate nature of SLBs, where flow in conventional microfluidic designs may interfere with established gradients, SLB, or cell migration. As such, conventional microfluidic models are not compatible with studying membrane-bound factors using SLBs.

Here, we engineered a multichannel device that orthogonally separates chemoattractant channels from SLB and cell loading channels by pillar-free, hydrogel barriers, to enable precise control of the timing and profile of chemokine gradients applied on cells interacting with SLBs. Using this model, we analyzed the synergistic effects of membrane-bound ICAM-1 and chemokine CXCL12 on Jurkat T cell migration.

## 2. Materials and Methods

### 2.1. Design, Fabrication, and Assembly of the Microfluidic Device

The design and toolpaths for the microdevice were created in Autodesk Fusion 360 (Autodesk, San Rafael, CA, USA) and were custom-milled (Shapeoko, Carbide 3D, Torrance, CA, USA) out of polycarbonate [26]. The final device was manufactured by pouring polydimethylsiloxane (PDMS) mixed in a 10:1 base to a curing agent ratio (Sylgard 184 elastomer kit; Dow Corning, Midland, MI, USA). PDMS was cured at 80 °C for 3 h, peeled off, and cut into individual devices. Channel inlets and outlets with 1.5 mm diameter were punched at both ends of microfluidic channels. The PDMS devices were permanently bound to the detergent-cleaned glass coverslips after plasma treatment for 50 s (Model PDC-001-HP, Harrick Plasma, Ithaca, NY, USA) for the subsequent lipid bilayer formation and substrate modification.

### 2.2. Formation of Gel Diffusion Barrier and Workflow of the Device

A solution of Poly-D-lysine (PDL) (VWR, Radnor, PA, USA) in Milli-Q^®^ water in the concentration of 0.5 mg/mL was injected into the two gel channels of a freshly assembled device, incubated at room temperature for 30 min, and aspirated with vacuum. 1× PBS was injected to the gel channels and aspirated out to remove excess PDL. A solution of agarose (Catalog number: 16500100; Invitrogen, Carlsbad, CA, USA) in the concentration of 0.8% wt/vol was freshly dissolved in water by microwaving for 45 s. Hot agarose solution was injected to the pre-coated hydrogel channels in the same fashion and allowed to solidify at RT for 30 min in a wet chamber to minimize hydrogel dehydration. After the formation of two gel barriers, lipid bilayer formation and/or protein capturing, cell seeding was performed in the center channel for chemotaxis studies (details described in corresponding method sections below).

### 2.3. COMSOL Simulation and Characterization of Transport Phenomena with FITC-Dextran

COMSOL Multiphysics^®^ (Stockholm, Switzerland) was used to simulate the gradient formation in the microdevice to guide the design of the gradient chamber geometry using the implemented Transport of Diluted Species in Porous Media Module. The porous media was approximated to be liquid water. Effective diffusivity model used was Millington and Quirk model. Diffusion coefficient of a 10 kDa molecule within the hydrogel and media was approximated to be 8.7 × 10^−11^ m^2^ s^−1^ and 9.25 × 10^−11^ m^2^ s^−1^, respectively [27]. Initial concentrations were set for the inlet and outlet reservoirs to represent the sink and the source, respectively (C = 0 and C = 5 × 10^−6^ mol/m^3^), with all other boundaries set to be “no flux”. All geometries in the model were defined with an extremely fine mesh in COMSOL Multiphysics. The model was then solved as a time-dependent study up to 120 min (time step = 5 min). For geometric parameter sweep, each of the five chambers of the device was generated as a rectangular solid with variable geometries (width, length and height). Simulated concentration gradients were obtained along a line that traversed the center cell chamber on the bottom surface, representing the concentration gradient experienced by cells seeded onto the membrane-bound or immobilized ICAM-1.

To experimentally demonstrate diffusion across the device, we loaded a solution of 10 kDa FITC-Dextran in PBS (2 μg/mL) into one reservoir chamber, PBS into the other chamber, and the center channel was filled with PBS to visualize the transport of fluorescent Dextran across the device by time-lapse imaging. Solutions of food colors were injected into the device, and the whole device was photographed to demonstrate the wall-less liquid confinement and diffusion across the platform.

### 2.4. Preparation of Supported Lipid Bilayers and Protein Tethered Surfaces

Lipid components, 18:1 (Δ9-Cis) 1,2-Dioleoyl-sn-glycero-3-phosphocholine (DOPC) and 5% 18:1 1,2-dioleoyl-sn-glycero-3-[(N-(5-amino-1-carboxypentyl)iminodiacetic acid)succinyl] (nickel salt) (DGS-NTA(Ni)), dissolved in chloroform, were purchased from Avanti Polar Lipids (Alabaster, AL, USA) and mixed. The lipids were dried in round-bottom flasks under a stream of N_2_ for 5 min and desiccated for 2 h with house vacuum pump in a chemical fume hood. The lipid mixture was resuspended by bath sonication in 1× PBS at a final concentration of 2.5 mg/mL and extruded 10 times through a membrane with 50 nm pore size (Avanti Polar Lipids, Alabaster, AL, USA) into small unilamellar vesicles (SUVs). The SUV solutions were then diluted 1:1 in 1× PBS (pH 7.4) before being loaded onto the detergent-cleaned and dried glass coverslip through the loading chamber, and incubated for 2 min to spontaneously form the lipid bilayers. The chambers were then washed with a 10× excess volume of 1× PBS.

### 2.5. ICAM-1 Capturing on Lipid Bilayer and Immobilization

For protein capturing on lipid bilayer, a solution of 10 µg/mL Alexa Fluor 568 labeled recombinant human ICAM-1 with poly-histidine and human Fc tag (Cat. 10346-H03H, SinoBiological, Beijing, China) was injected onto SLB, incubated at RT for 40 min and tethered to 18:1 DGS-NTA(Ni) through chelation. Tethered SLB was washed excessively with 1× PBS before use. For the immobilization of ICAM-1, 100 µg/mL recombinant protein A (Cat.101100, Thermo Fisher Scientific, Waltham, MA, USA) in 1× PBS was injected onto a detergent-cleaned and dried glass coverslip, incubated for 30 min, washed with 1× PBS, before the same solution of human ICAM-1 was injected and incubated at RT for 40 min. The resulted substrate was then washed with 1× PBS before use.

### 2.6. Cell Seeding and Incubation

Jurkat cells were cultured in ATCC-formulated RPMI-1640 Medium (Cat. ATCC 30-2001, ATCC, Manassas, VA, USA) supplemented with 10% Fetal Bovine Serum (FBS) (Cat. F2442, Sigma-Aldrich, St. Louis, MO, USA) and 1% 100 U/mL penicillin-streptomycin (Sigma-Aldrich, St. Louis, MO, USA). Cells were sub-cultured every 2–3 days and kept in sterile incubation conditions (37 °C, 5% CO_2_ and 90% humidity) according to ATCC protocols. Cells were labeled with Calcein AM (Cat. C1430, Thermo Fisher Scientific, Waltham, MA, USA) according to the manufacturer’s protocol before being loaded onto the device at a final density of 150 cells/mm^2^. The chamber containing lipid bilayers was equilibrated with the same media, and the resuspended cells were then injected into the chamber and incubated for 1 h in a humidified incubator maintained at 37 °C and 5% CO_2_. Cell culture media with or without 50 ng/mL of CXCL12 (Cat. 250-20A, PeproTech, Cranbury, NJ, USA) was injected into the two reservoirs of the device, respectively. Then, cells were imaged at 2× at an interval of 5 min, for a total period of 1 h for chemotaxis analysis.

### 2.7. Imaging, Cell Tracking, and Data Analysis

A Nikon Eclipse Ti-E inverted fluorescence microscope (Nikon, Tokyo, Japan) was used for live-cell imaging, equipped with an Okolab incubation box (Okolab, Pozzuoli, Italy) controlling for temperature (37 °C) and CO_2_ concentration (5%). Images were taken using a 2× objective (CFI60 Plan Apochromat Lambda, NA 0.1, Nikon, Tokyo, Japan). Images were analyzed using ImageJ (U.S. National Institutes of Health, Bethesda, MD, USA; http://rsb.info.nih.gov/ij (accessed on 1 August 2021)). Two open-source plugins, “Manual Tracking” and “Chemotaxis and migration tool 2.0”, along with customized MATLAB (MathWorks, Natick, MA, USA) codes, were used to analyze time-lapse images and cell tracking data.

### 2.8. Statistics

All experiments were repeated at least three times. All data are presented in mean ± SD. n represents cell number analyzed in each experiment, as detailed in figure legends. One-way ANOVA or two-tailed Student’s *t*-tests were used for evaluating the significance of difference unless otherwise indicated. Statistical analyses were performed using GraphPad Prism 7 software (GraphPad Software, San Diego, CA, USA). Not specified: *p* > 0.05; *: *p* < 0.05, ***: *p* < 0.001, ****: *p* < 0.0001.

## 3. Results

### 3.1. A Multichannel Device Design Allows for Separate Lipid Bilayer and Chemoattractant Gradient Formation

**Figure 1:**
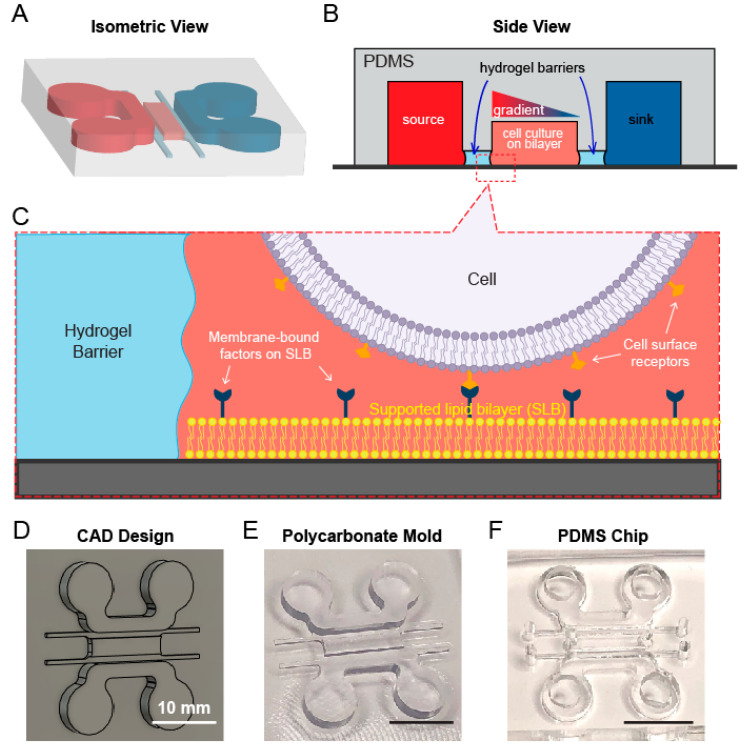
Design of a microchannel diffusion device for chemotactic studies. (**A**) An isometric view of the device design. (**B**) Schematics of the cross section of microchannels. (**C**) A zoomed-in illustration of cell culture chamber. (**D**) CAD design of the PC mold. (**E**) A micro-milled PC mold. (**F**) A PDMS device replica-molded from the PC mold and drilled with inlets and outlets.

To study the role of membrane-bound interactions in cell chemotaxis, we designed a multichannel microdevice that contains both a chemoattractant gradient and a lipid bilayer for chemotactic migration under the context of membrane-bound factors and interactions (Figure 1A). The device is geometrically symmetrical and composed of five channels: a center channel for cell culture, two large reservoir channels serving as the source and sink of chemoattractants, and two thin hydrogel barrier channels that separate the lipid bilayers and cell culture from the reservoir channels (Figure 1A,B). The five channels differ in heights and are laterally connected. The chemoattractant gradient was established across the width of the central channel by the diffusion of soluble factors from source to sink channel (Figure 1B). Two hydrogel barriers were permeable to chemoattractants but not cells, allowing for independent handling of lipid bilayer formation and cell loading from the gradient generation (Figure 1C). Typical microfluidic gel barriers often involve micropillar structures to hold gel in the channels [28,29,30], which however can lead to non-uniform chemoattractant distribution in the central channel due to the blockade of diffusion. To avoid this, we employed a pillarless, liquid pinning strategy [31] which utilizes the capillary force and surface tension to draw and hold gel solution in the barrier channels, thus allowing for a simplified design of gel barrier channel without interfering with lateral diffusion profiles (Figure 1B). We then carried out a proof-of-concept fabrication workflow for the multichannel device. A master mold of the device was designed in Autodesk Fusion 360 (Figure 1D) and milled in polycarbonate (PC) on a Carbide 3D Nomad desktop milling machine (Figure 1E). The device was then replica-molded in polydimethylsiloxane (PDMS), drilled with inlets and outlets with biopsy punches to allow for downstream studies (Figure 1F).

### 3.2. Channel Height and Surface Treatment Are Key to Liquid Pinning-Based Hydrogel Barrier Formation

**Figure 2:**
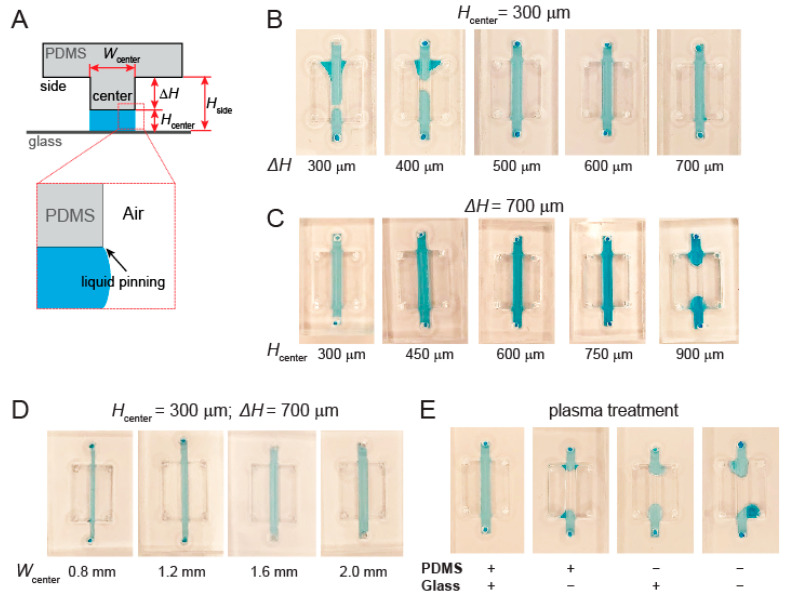
Dependence of liquid pinning on device parameters and surface treatment. (**A**) Illustration of key geometric parameters for liquid pinning. (**B**) Varying the height difference between the center and side channel with the height of the center channel kept constant (300 µm). (**C**) Varying the height of the center channel with the height difference kept constant (700 µm). (**D**) Varying the width of center channel with constant height of the center channel (300 µm) and the height difference (700 µm). (**E**) Effect of plasma treatment of the PDMS device and glass substrate on liquid pinning.

The hydrogel barriers are a crucial component for separating the lipid bilayer/cell culture channel from the chemoattractant channels in our device. We first investigated the design parameters of the hydrogel channels that are key to their ability to pin liquids in order to form the hydrogel barriers. To illustrate this concept, a simplified version of the hydrogel barrier channel was designed, which contains a central liquid channel flanked by two taller air channels on both sides for liquid pinning (Figure 2A). The lateral dimensions of the center and side channels were designed as 2 mm × 20 mm and 4 mm × 12 mm, respectively. We tested liquid pinning on the center channel on different device designs, using water with blue food coloring for visualization. The success of liquid pinning was defined as the retention of the aqueous solution in the center channel without breakage or spillage into either of the side channels.

The first geometric parameter we examined was the difference in the heights of center and side channels, which helps restrain the vertical advancement of liquid–air interface into the side channels (Figure 2A). With the height of center channel fixed at 300 µm, we varied the height of side channels so that the height difference (ΔH) varied from 300~700 µm at a 100 µm step (Figure 2B). We found that a minimum height difference of 500 µm was required to pin liquid in the center channel (Figure 2B). Next, we held ΔH at 700 µm while varying the height of the center channel (H) from 300 to 900 µm at a 150 µm step (Figure 2C). Our test results showed that the design was able to support liquid pinning in a wide range of center channel heights up to 750 µm (Figure 2C). We further examined whether the width of the center channel also plays any role in liquid pinning. With fixed H at 300 µm and ΔH at 700 µm, we found that all the tested widths (from 0.8 mm to 2.0 mm) successfully achieved liquid pinning (Figure 2D). These results suggest that liquid pinning in the hydrogel channel is dependent on and sensitive to the height and height difference but not the width of the channels in our device design.

Another crucial factor in liquid pinning is the hydrophobicity of the microchannel surfaces, which determines the surface tension that enables microchannel wetting and retention of the aqueous solution in the center channel during the pinning process. Plasma treatment is a common step in microfluidic device fabrication, which covalently binds PDMS to glass while reducing the hydrophobicity of the internal surfaces, particularly for those of PDMS [32]. While all the devices tested so far had been treated with oxygen plasma (under atmospheric conditions), we next specifically examined the impact of such treatment on the ability of achieving liquid pinning in the device (Figure 2E). Using a design of H at 300 µm and ΔH at 700 µm, we evaluated the liquid pinning in the devices with plasma treatment on neither, either, or both PDMS and glass surfaces before device assembly (Figure 2E, bottom). We found that liquid pinning was successful only when both PDMS and the glass coverslip were treated. Therefore, plasma treatment is necessary not only for device assembly but also for liquid pinning.

### 3.3. Coating Hydrogel Channel Is Necessary to Prevent Leakage of Soluble Factors

The key design of the device lies in the proper functions of hydrogel barrier channels. Ideally, a hydrogel barrier should form a good seal around the PDMS/glass interfaces, allowing for chemoattractant diffusion through the barrier while preventing cells from escaping the bilayer/cell culture channel. It should also maintain shape within the channel throughout the whole process of lipid bilayer formation, protein tethering, cell seeding and live imaging (Figure 1B,C).

**Figure 3:**
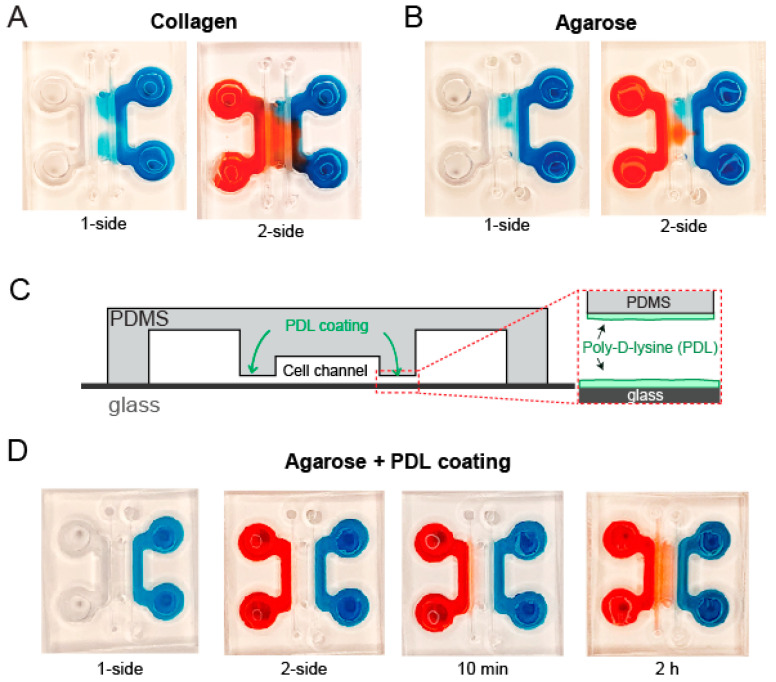
Effect of hydrogel type and poly-D-lysine precoating. Leakage of dye solution into the center channel when the hydrogel barriers were formed with (**A**) collagen and (**B**) agarose hydrogel without precoating in the device. (**C**) Illustration of poly-D-lysine (PDL) precoating in the gel channels. (**D**) PDL precoating prevented leakage in the agarose hydrogel barrier.

After an initial screening, we narrowed down to two hydrogel candidates, collagen and agarose, which maintain their shape in the hydrogel barrier channels (Figure 3A,B). The hydrogel solution of selection (2.5 mg/mL collagen solution or a 0.8% agarose solution) was injected into corresponding channels and allowed to be cured or solidified. To test the diffusivity and integrity of the gel barriers, we injected aqueous solutions with blue and red food dyes into one or both reservoir (source and sink) channels and monitored the distribution of the colors across the hydrogel barrier for 2 h. We found that the blue and red food dyes immediately appeared across the hydrogel barriers and in the center channel in an uncontrollable manner (Figure 3A,B), suggesting leakages at the gel–PDMS or gel–glass interfaces.

Poly-lysine is a positively charged synthetic polymer of the amino acid(s) L-lysine or D-lysine. It has been widely used as an enhancer of electrostatic interactions for surface coating [33]. On the other hand, the agarose polymer contains negatively charged residues, namely pyruvate and sulfate [34]. We thus hypothesized that pre-coating the channel surfaces with poly-lysine would electrostatically seal the gel–PDMS and gel–glass interfaces. We coated the hydrogel channel with poly-d-lysine (PDL) prior to injecting and solidifying a 0.8% agarose hydrogel, followed by infusion of aqueous food dye solutions to the reservoir channels. We indeed observed a steady retention of the dye solutions in the side channels, which uniformly diffused across the agarose hydrogel barrier during the 2 h incubation period (Figure 3C). Considering the superior performance of the agarose hydrogel and the concern of collagen as an adhesion substrate, we decided to use agarose gel with PDL precoating for the following chemotaxis studies.

### 3.4. Gradient Profiles within the Device Can Be Optimized through COMSOL Simulation

**Figure 4:**
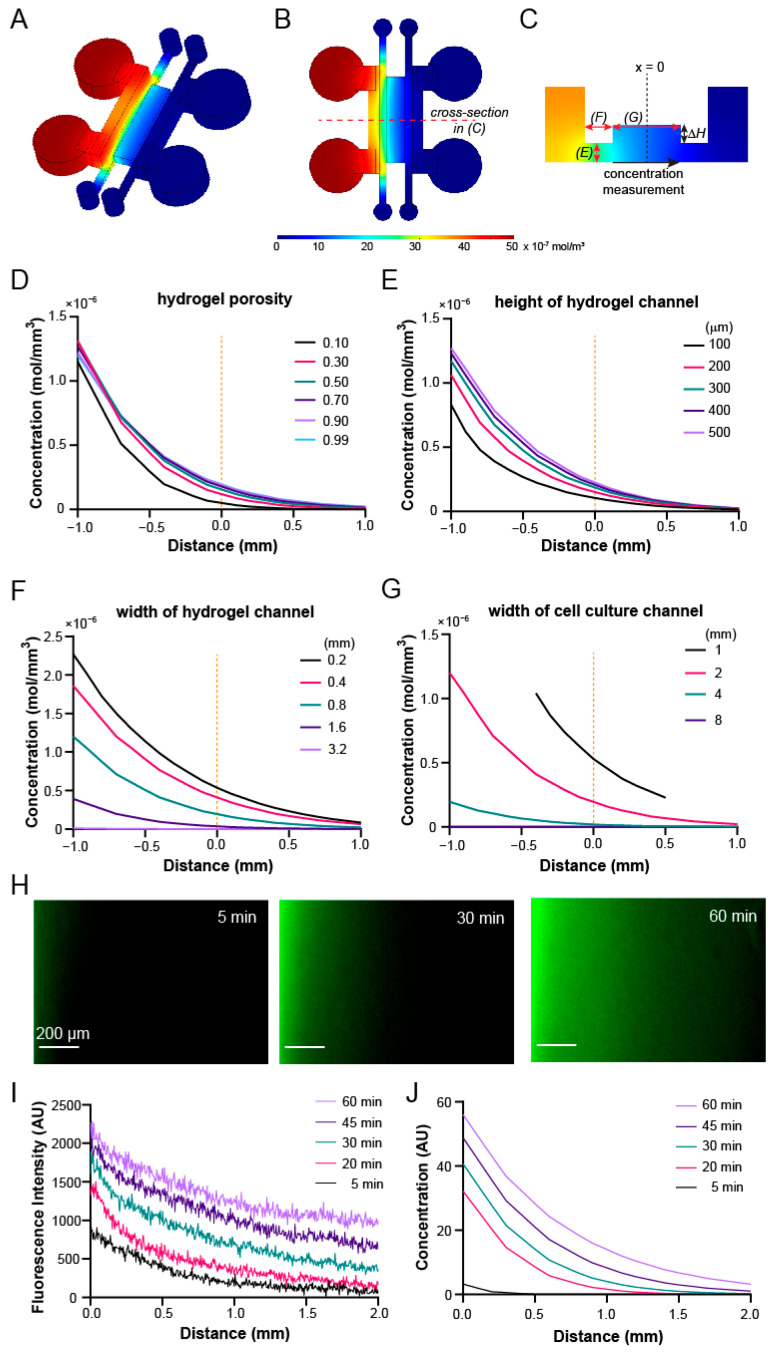
Characterization of diffusion with COMSOL Multiphysics^®^ and FITC-Dextran diffusion. (**A**) Isometric, (**B**) top, and (**C**) cross-sectional views of concentration distribution throughout the diffusion microdevice in COMSOL simulation. (**D**–**G**) COMSOL parameter sweep results of the effects of key geometries on the concentration profiles across the center channel. Default parameters during sweep include width of the cell chamber (2 mm), width of the hydrogel chamber (0.8 mm) and height of the hydrogel chamber (0.35 mm). Simulated effect of (**D**) hydrogel porosity, (**E**) height of hydrogel barrier, (**F**) width of hydrogel channel, and (**G**) width of the center SLB/cell loading channel. (**H**) Diffusion of 10 kDa FITC-Dextran in center chamber over time. (**I**) Quantification of fluorescence intensity of 10 kDa FITC-Dextran across the width of the center channel over time. (**J**) Simulated concentration profiles of 10 kDa molecule across the center channel over time.

Upon confirming the requirements of design parameters and feasibility of the hydrogel barrier, we next utilized COMSOL Multiphysics simulation to determine the optimal device parameters for the chemotaxis studies (Figure 4A–C). The criteria for an ideal device include a steep chemokine gradient and a wide migration space in the cell culture channel, and compatibility with the milling- and liquid pinning-based fabrication/assembly processes. Specifically, we used the simulated concentration profile across the width of the lipid bilayer/cell culture channel at 1 h as the readout (Figure 4C), as it is where the cells will be seeded and migrate. The first parameter that we examined was the hydrogel porosity, defined as volume ratio between pores and total bulk, which varies between 0 (no pores) and 1 (fully liquid). As a reference, the porosity of commonly used agarose gel of low concentration (<1%) has a porosity close to 1. We found that the concentration distribution is similar when the porosity varies in the range of 0.1–1 (Figure 4D), suggesting that gel porosity is not a critical factor for the gradient profile within the device.

Next, we screened the geometric parameters of the hydrogel barrier and central channel (Figure 4C, red arrows). We fixed the height of the central lipid bilayer/cell culture channel at 1 mm to allow for a sufficient height difference (ΔH) between the hydrogel barrier and central channel for liquid pinning (Figure 2B and Figure 4C). We first examined the impact of the height of the hydrogel barrier on the gradient profile within the central channel, by varying the height of hydrogel barrier from 100 to 500 µm (thus ΔH from 900 to 500 µm). We found that the gradient profiles were largely similar under the heights of 300 and 500 µm, while the steepness of the gradient near the center dropped more significantly under the 100 and 200 µm heights (Figure 4E). We then evaluated the impact of hydrogel barrier width on the gradient profile, by varying its value from 0.2 to 3.2 mm. The simulation showed that the gradient was quickly flattened by the increased width above 0.4 mm (Figure 4F), suggesting a shift of major diffusion resistance from the central channel to the hydrogel barriers under the increased gel barrier widths. Lastly, we evaluated the impact of the central channel width on the gradient profile (Figure 4G). While reducing the channel width from 2 mm to 1 mm slightly improved the steepness of the gradient at the channel center, increasing it to 4 mm and 8 mm dramatically flattened the chemoattractant gradient (Figure 4G). Combining these results and the choice of milling tool size (1/32”), to achieve liquid pinning as well as a steep gradient with ease of fabrication, we finalized a design with a 350 µm hydrogel channel height, a 0.8 mm hydrogel channel width, and a 2 mm central channel width.

With these chosen parameters, we fabricated a microchannel diffusion device and characterized the spatiotemporal gradient profiles of 10 kDa fluorescence isothiocyanate (FITC)-dextran, which serves as a surrogate for the chemoattractant CXCL12 [35]. Fluorescence images were taken every 5 min for 1 h upon the filling of the source channel with FITC-dextran (Figure 4H). We observed a series of time-dependent lateral concentration gradients within the central channel (Figure 4I), which was overall consistent with those from COMSOL simulations (Figure 4J). Overall, we designed and optimized the device based on COMSOL simulation and demonstrated the establishment of a concentration gradient within the device for subsequent chemotaxis studies.

### 3.5. Fluorescence Recovery after Photobleaching Confirms Lipid Bilayer Formation and Mobility in the Device

**Figure 5:**
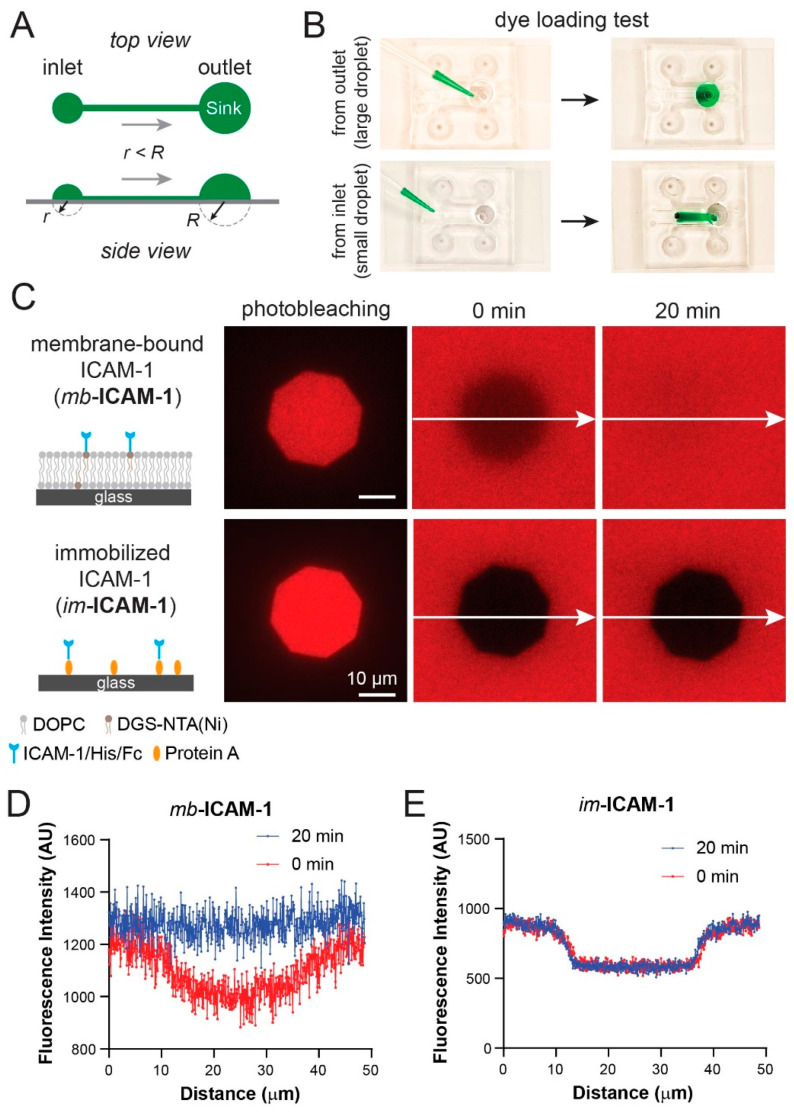
Lipid bilayer formation and confirmation of lateral mobility of the membrane-bound ICAM-1. (**A**) Schematic of filling the center channel with Laplace pressure. R and r are the radii of the larger and smaller droplets, respectively. The difference in pressure generated by the surface tension of each droplet drives liquid movement in the microchannel. (**B**) Dye loading test showing the filling direction. (**C**) Evaluating lateral mobility of membrane-bound (mb-) and immobilized (im-) ICAM-1 with fluorescent recovery after photobleaching (FRAP). (**D**,**E**) Line scan of mb-ICAM-1 and im-ICAM-1 fluorescence profiles right after vs. 20 min after photobleaching.

Next, we tested whether a lipid bilayer can be formed and tethered with membrane-bound proteins in the microdevice. To avoid excessive shear flow during the loading and washing steps in the center channel, which may disrupt lipid bilayer/tethered proteins/attached cells, we used the difference in Laplace pressures generated by the curved liquid–air interface droplets at the inlet and outlet of the center channel [36] to inject samples (liposome solution, membrane-bound proteins, and cells) in the device (Figure 5A). We first established the agarose hydrogel barriers in the flanking channels, before filling the center channel with aqueous solution. We intentionally overfilled the channel so that it formed a large droplet at the outlet and a small droplet at the inlet. The formation of the droplets indicated no leakage through the hydrogel barrier. To demonstrate the sample loading direction and assess the uniformity of loaded solution, we pipetted 50 µL of green food dye solution to the outlet (large droplet) or inlet (small droplet), respectively. Only the dye solution loaded at the inlet (small drop) flowed through the whole channel with uniform distribution across the width of the channel (Figure 5B), confirming the feasibility of the Laplace pressure-based sample loading.

We then injected an aqueous solution containing small unilamellar vesicles (SUVs) composed of synthetic lipid 1,2-Dioleoyl-sn-glycero-3-phosphocholine (DOPC) mixed with 5% 1,2-dioleoyl-sn-glycero-3-[(N-(5-amino-1-carboxypentyl)iminodiacetic acid)succinyl] (nickel salt) (DGS-NTA(Ni)) lipid into the center channel of a prepared device to form an SLB. We loaded 6-histidine- and Fc-tagged, fluorescently-labeled intercellular adhesion molecule-1 (ICAM-1) to the channel to tether ICAM-1 to SLB through Ni-chelation. As an immobilized control, we also coated a channel with Protein A and immobilized the same ICAM-1 through the capture of Fc domain by the Protein A layer (Figure 5C). Fluorescence recovery after photo-bleaching (FRAP) technique was used to confirm the lateral mobility (or immobility) of the captured ICAM-1 on both surfaces, in which a small region within the channel was photobleached and monitored for fluorescence recovery for 20 min (Figure 5C). The fluorescence recovery was observed immediately upon photobleaching on the SLB (Figure 5C, top row, mb-ICAM-1; Figure 5D), while, in contrast, no recovery was observed with the immobilized ICAM-1 (im-ICAM-1) (Figure 5C, bottom row; Figure 5E). Therefore, our device is capable of forming substrates with membrane-bound factors and immobilized factors.

### 3.6. Jurkat Cells Have Different Chemotactic Profiles on Membrane-Bound vs. Immobilized ICAM-1

**Figure 6:**
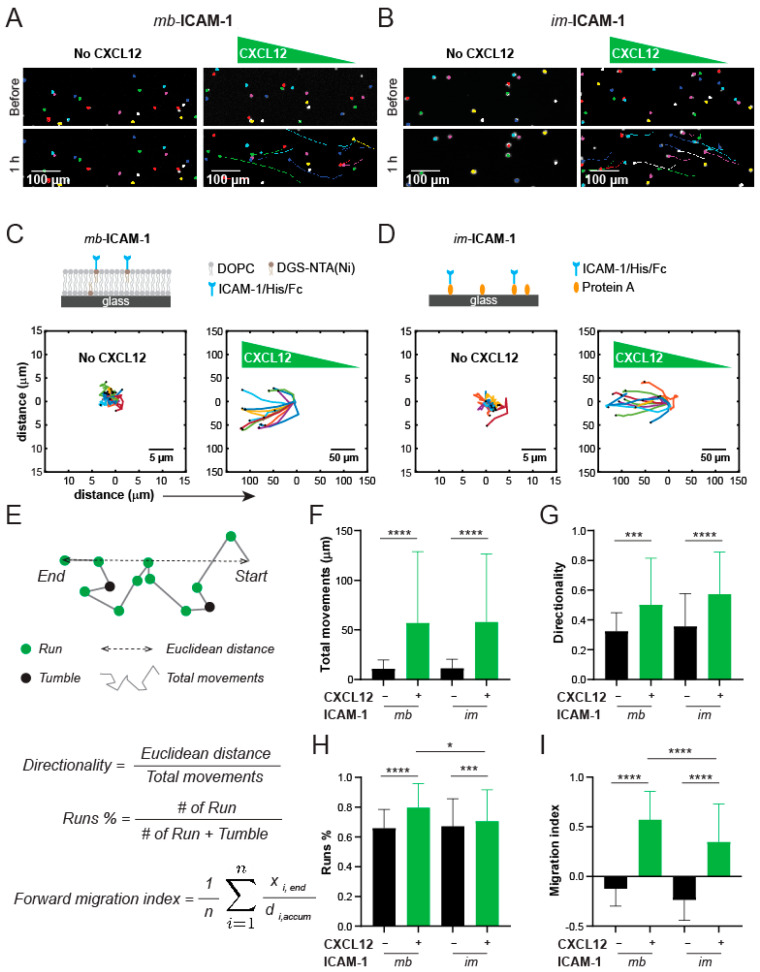
Jurkat cell chemotaxis towards CXCL12 on membrane-bound vs. immobilized ICAM-1 in the diffusion microdevice. Representative images of Jurkat cells and migration trajectories in 1 h with and without CXCL12 gradient, on (**A**) mb-ICAM-1 and (**B**) im-ICAM-1, respectively. The migration trajectories of Jurkat T cells in 1 h on (**C**) mb-ICAM-1 and (**D**) im-ICAM-1 without and with CXCL12 gradient. (**E**) Schematic of the definitions of cell migration parameters. Quantification of (**F**) total movements, (**G**) directionality, (**H**) migration persistency as assessed by Runs% and (**I**) forward migration index, as assessed by averaged movement toward chemoattractant gradient over accumulated distance. *n* = 66–71 single cell trajectory per condition. Not specified: *p* > 0.05; *: *p* < 0.05; ***: *p* < 0.001; ****: *p* < 0.0001 by ANOVA with Tukey’s test.

As a proof-of-concept, we next evaluated the chemotactic behaviors of Jurkat T cells on mb-ICAM-1 vs. im-ICAM-1 under a gradient of CXCL12 within the device. After forming the hydrogel barriers, we first loaded the devices with either SLB with tethered ICAM-1, or immobilized ICAM-1 through a coated Protein A layer in the center channel. Jurkat cells were then seeded on the two substrates and incubated for 1 h to allow for initial cell attachment. To form a chemokine gradient, we loaded the two reservoir channels with media containing 50 ng/mL CXCL12 or no CXCL12, respectively. Cells were immediately live imaged at an interval of 5 min for a total period of 1 h side-by-side on both surfaces (Figure 6A,B). Noticeably, we observed little or no cell movement in the control devices without CXCL12 gradient, whether on the membrane-bound or immobilized ICAM-1 (Figure 6C,D, left panels; scale bars: 5 µm). In contrast, CXCL12 gradient induced massive and persistent migration toward the sources of the gradient in the Jurkat cell population on both substrates (Figure 6C,D, right panels; scale bars: 50 µm). To assess the migratory behaviors of the Jurkat cells, we tracked the trajectory of the cell movement on the substrates, and quantified the total movements, directionality, percent of runs, and the forward migration index along the *x*-axis (Figure 6E). Among those, total movements indicate the level of migratory activities, while directionality indicates the randomness of migration. The “runs” are defined as the movement along the overall migration direction, while “tumbles” are those backward movements. The percentage of runs thus reflects the responsiveness of migration to the chemoattractant source. The forward migration index (on the *x*-axis) is a measure of the efficiency of directed migration toward the chemoattractant. We observed that under both surface conditions, Jurkat cells had significantly higher total movements and directionality under chemoattractant gradients than those without the gradient (Figure 6F,G). A detailed analysis on the percentage of runs and forward migration index further showed that those Jurkat cells migrating on mb-ICAM-1 on SLBs had higher responsiveness and deficiency in their directed migration toward the chemoattractant source than their counterparts on the im-ICAM-1 (Figure 6H,I). Our results thus demonstrated the ability of our device to investigate cell chemotaxis and distinguish the differential behaviors of cell migration on membrane-bound vs. immobilized factors.

## 4. Discussion

We developed a cleanroom-free, multifactor device which is, to our knowledge, the first attempt designed to study cell chemotaxis on a cell membrane-mimicking lipid bilayer. We studied the geometry dependence of liquid pinning and the effect on gradient generation within the device, which can be utilized to guide and inspire microfluidic device design. Admittedly, the milling strategy also imposed a limit on the smallest features of the microchannel designs, such as by the size of the drill bits and the spatial resolution (x, y, and z) of the milling platform. As such, the sharpest gradient indicated in the COMSOL simulation was not achieved. Nevertheless, the low price of and ease of access/operation to milling platforms will allow for wide adoption of this platform for small labs with insufficient resources or those with limited access to cleanroom facilities. On the other hand, we can further improve the sharpness of gradients within the device using a milling platform with higher resolution/smaller tools by reducing the width of gel barrier, and improve the uniformity/longevity of the gradients by increasing the size of reservoirs.

Notably, in the last decade, several groups have created devices for chemotaxis studies using Jurkat T cells as a model [37,38,39,40]. While none of them were constructed for chemotactic studies on SLBs, some of their characteristics concur with ours, such as the gel channels [37], the flow-free design [40] or the utilization of micro-milling [38]. Some design features may be integrated in ours to further improve its functionality. For example, Amadi et al. incorporated low resistance channels to dissipate pressure gradients between source and sink channels, which eliminates interstitial flow through the hydrogel channels and significantly improved the longevity of the gradients [37]. Pietrosimone et al. utilized an electric cell-substrate impedance sensing (ECIS) system [39] to assess cell migration by an increase of resistance as cells pass over the electrodes [41]. Coluccio et al. took advantage of a gravity-driven flow in the source/sink channels to improve the stability of chemotactic gradient [38]. Sonmez et al. integrated a porous membrane to separate the cell migration chamber from the source/sink channels [40], which drastically shortens the diffusion distance between the source/sink and cell migration chambers. We can foresee our platform incorporating some of these features individually or in combination to further improve the quality and/or longevity of the gradient (e.g., low resistance channels, membrane barrier, or flow in source/sink), or the ease of the result readouts (e.g., ECIS).

Our platform can be easily adapted and extended to different biological contexts since each channel within the device can be manipulated independently. By coating the center chamber differently (ECM proteins, immobilized proteins, cultured cells, etc.), cell adhesion and migration can be studied accordingly. Substrate rigidity can affect the speed of cell migration [42]. Instead of the rigid glass substrate, we can alternatively form an SLB on a hydrogel with tunable mechanical behavior in the central channel [43], to recapitulate the mechanical properties of stromal cells. Furthermore, shear flow can be included as another factor by connecting the center channel to a syringe pump, to study cell migration under a controlled shear stress. In addition, by changing the soluble molecule content in the two reservoirs, cell responses to more soluble factors can be studied simultaneously. Since the migration of many cell types is achieved through cell–cell adhesion and chemokines in the microenvironment, our platform can be widely adapted to different biological contexts, such as membrane-bound migration in cancer, immune, and stem cells.
